# Supplementary material for: Causal Relationship between Aging and Anorexia Nervosa: A White-Matter-Microstructure-Mediated Mendelian Randomization Analysis
Source: Biomedicines. 2024 Aug 16;12(8):1874. doi: 10.3390/biomedicines12081874 (PMC11351342; doi:10.3390/biomedicines12081874)
Supplement: Supplementary file 1 [file biomedicines-12-01874-s001.zip › Supplementary Figure/Figure S9/Leaveoneout plot of MR analysis for ubm-b-1455 to AN.pdf]

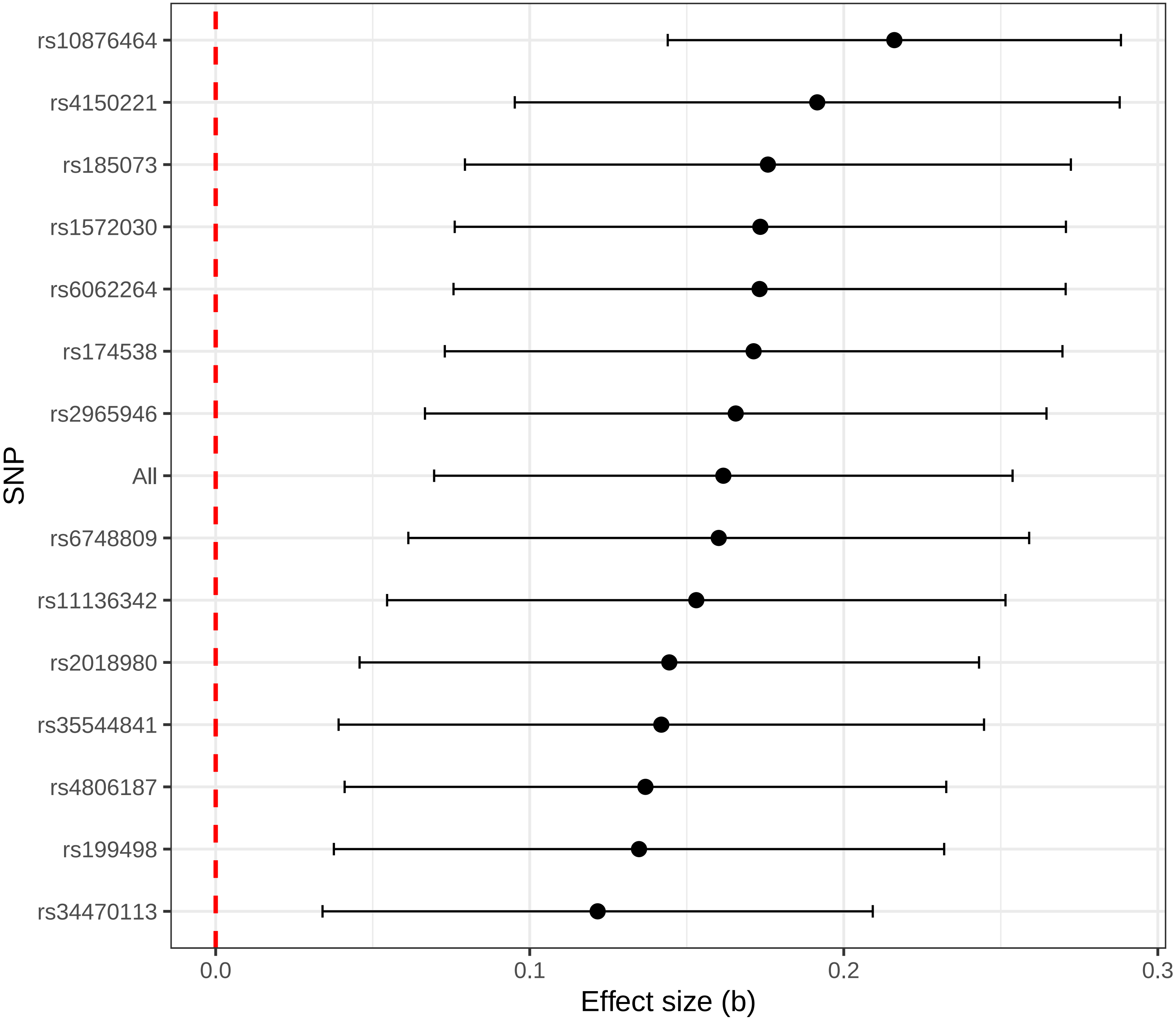
rs10876464
rs4150221
rs185073
rs1572030
rs6062264
rs174538
rs2965946
All
rs6748809
rs11136342
rs2018980
rs35544841
rs4806187
rs199498
rs34470113
SNP
0.0
0.1
0.2
0.3
Effect size (b)
